# The *h*-index as an almost-exact function of some basic statistics

**DOI:** 10.1007/s11192-017-2508-6

**Published:** 2017-09-09

**Authors:** Lucio Bertoli-Barsotti, Tommaso Lando

**Affiliations:** 1 Department of Management, Economics and Quantitative Methods, University of Bergamo, Via dei Caniana 2, 24127 Bergamo, Italy; 2 Department of Finance, VŠB -TU Ostrava, Sokolskà 33, 70121 Ostrava, Czech Republic

**Keywords:** *h*-Index, Journal ranking, Weibull distribution, Lambert *W* function, 62P99, C46

## Abstract

As is known, the *h*-index, *h*, is an exact function of the citation pattern. At the same time, and more generally, it is recognized that *h* is “loosely” related to the values of some basic statistics, such as the number of publications and the number of citations. In the present study we introduce a formula that expresses the *h*-index as an almost-exact function of some (four) basic statistics. On the basis of an empirical study—in which we consider citation data obtained from two different lists of journals from two quite different scientific fields—we provide evidence that our ready-to-use formula is able to predict the *h*-index very accurately (at least for practical purposes). For comparative reasons, alternative estimators of the *h*-index have been considered and their performance evaluated by drawing on the same dataset. We conclude that, in addition to its own interest, as an effective proxy representation of the *h*-index, the formula introduced may provide new insights into “factors” determining the value of the *h*-index, and how they interact with each other.

## Introduction

The purpose of this paper is to present a formula with which to determine (estimate) the *h*-index, *h*, under incomplete information conditions (IIC). By IIC we mean the situation in which, for different kinds of reasons, we do *not* know the whole set of citation data, the entire citation profile that would allow us to obtain the actual exact value of the *h*-index. This is the case, for example, when only few “basic” citation statistics (other than the *h*-index) are published, or known to us.

To be concrete, we will refer to simple citation indicators—to use the words of Hirsch (2005), “single-number criteria commonly used to evaluate scientific output”—as:

1. total number of citations $C$;
2. total number of citations for the $t$ ($t \in \left\{ {1,2,3, \ldots } \right\}$) most-cited publications, $C_{t}$; thus, $C_{t} = \sum\nolimits_{i = 1}^{t} {c\left( i \right)}$, where $c\left( i \right)$ represents the number of citations to publication *i*, and where publications are ranked in decreasing order of the number of citations: $c\left( 1 \right) \ge c\left( 2 \right) \ge \cdots \ge c\left( T \right)$.
3. total number of publications $T$;
4. total number of “significant” publications, that is, those with at least a predetermined number of citations $k$ each ($k \in \left\{ {1,2,3, \ldots } \right\}$), $T_{k}$.

In this paper we focus on these indicators in their simplest versions, that is: $C$, $C_{1}$, $T$ and $T_{1}$. The purpose of the analysis is twofold: to estimate the *h*-index (when it cannot be determined directly from the data) and hence at the same time to identify the main factors which influence the level of the *h*-index. A crucial question is therefore the extent to which the *h*-index can be satisfactorily predicted from knowledge of only the above basic statistics—i.e. under IIC.

More formally, we are searching for a formula

1 $$\hat{h} = \hat{h}\left( {S_{1} , \ldots ,S_{r} } \right),$$

$1 \le r \le 4$, $S_{j} \in {\mathcal{S}}$, $1 \le j \le r$, where ${\mathcal{S}} = \left\{ {C,C_{1} ,T,T_{1} } \right\}$. To be noted is that the formula $\hat{h}$ can be interpreted as a *genuine* estimator of the *h*-index, $h$, i.e. $\hat{h} \cong h$, because it does not depend on values of unknown parameters.

Possible estimators under IIC of the *h*-index can be found in the literature:

- A very simple proxy for the *h*-index is given by $h_{H} = \sqrt {C/a}$. This model, which can be traced back to Hirsch (2005), is not a genuine estimator of the *h*-index because $h_{H}$ is still a function of an unknown parameter, $a$, and it is not specified (by the formula itself) how to estimate this parameter in terms of the above basic statistics. Nevertheless, an estimator for the *h*-index can be obtained by substituting the unknown parameter $a$ with a fixed constant (Hirsch found “empirically” that $a$ lay between 3 and 5). Redner (2010) found that “$\sqrt C$ is essentially equivalent to the *h*-index, up to an overall factor that is close to 2” (put otherwise, he found that the distribution ratio $\sqrt C /2h$ has an empirical distribution “sharply peaked about 1”). This suggests the approximating formula
  2 $$\hat{h} = h_{R} = \sqrt C /2$$
  with $r = 1$, ${\mathcal{S}} = \left\{ C \right\}$, which we could then call the *Redner formula*—probably the simplest estimator of the *h*-index, under IIC.
- While $h_{R}$ is a *model*-*free* proxy for the *h*-index, more elaborate solutions has been attempted in the literature by assuming specific probabilistic distributions for the citation rate. For example, a formula that follows model (1), with $r = 4$, has been recently introduced by Bertoli-Barsotti and Lando (2017),
  3 $$\hat{h} = \tilde{h}_{W}^{\left(1 \right)} = \frac{- 1}{{\log \left({1 - \tilde{m}_{1}^{- 1}} \right)}} \cdot W\left({\frac{{T_{1}}}{{1 - \tilde{m}_{1}^{- 1}}} \cdot \log \left({1 - \tilde{m}_{1}^{- 1}} \right)} \right),$$
  where $\tilde{m}_{1} = \left( {C - C_{1} } \right)/\left( {T_{1} - 1} \right)$ is nothing but a “trimmed” version of the simple sample mean $C/T_{1}$, and where $W\left( \cdot \right)$ represents the so-called Lambert-*W* function (Corless and Jeffrey 2015). The Lambert-*W* function is the function $W\left( z \right)$ satisfying $z = W\left( z \right){\text{e}}^{W\left( z \right)}$, and can be currently computed using mathematical software, for example the Mathematica^®^ software package (Wolfram Research, Inc. 2014), or the R statistical computing environment (R Development Core Team 2012). The use of a “trimmed” version of the sample mean is a simple technique with which to make the sample mean more robust with respect to a single outlier—a single highly-cited paper that could substantially inflate the mean, as is well known.

Formula $\tilde{h}_{W}^{\left( 1 \right)}$ $(r=4,\,{\mathcal{S}} = \left\{ {C,C_{1} ,T,T_{1} } \right\})$ is based on the assumption that the citation rate of papers (cited at least once) follows a *shifted*-*geometric distribution* (SGD) with parameter $Q(Q>1)$ with probability function $p\left( y \right) = Q^{ - y} \left( {Q - 1} \right)^{y - 1}$, $y = 1,2, \ldots$; $p\left( y \right)$ represents the probability of observing the number of citations $y$ of a paper (cited at least once), while $Q$ represents the expectation of the SGD. Then, $\hat{n}\left( y \right) = Tp\left( y \right)$ expresses the “expected”/estimated number of articles with $y$ citations.

- As an alternative approach, an important class of models is the one defined by the formula
  4 $$\hat{h} = \gamma_{0} C^{2/3} T^{ - 1/3}$$
  where $\gamma_{0}$ is a fixed and *known* positive constant (Schubert and Glänzel 2007). From model (4), specific ready-to-use formulas are obtained by taking, in particular: (a) $\gamma_{0} = 4^{ - 1/3}$ (Iglesias and Pecharroman 2007; see also Ionescu and Chopard 2013; Panaretos and Malesios 2009; Vinkler 2009, 2013), (b) $\gamma_{0} = 0.75$ (Schubert and Glänzel 2007), (c) $\gamma_{0} = 1$ Prathap (2010a, b). Following the notation of Bertoli-Barsotti and Lando (2017), let $h_{SG} \left( {\gamma_{0} } \right) = \gamma_{0} C^{2/3} T^{ - 1/3}$. Note that these formulas are functions of the data only through two out of the four basic statistics ($r = 2$, ${\mathcal{S}} = \left\{ {C,T} \right\}$), and they are based on the assumption of a *continuous*-type distribution. The formula $h_{SG} \left( 1 \right)$ is also known as the “*p*-index” (Prathap 2010a, b).
- Another approach which deserves mention for completeness, even if it does not yield a ready-to-use formula, is that proposed by Iglesias and Pecharroman (2007). Adopting a different perspective, i.e. the rank-size formulation, and starting from the assumption that the number $c\left( k \right)$ of citations of the paper of rank $k$, is approximately distributed following a *stretched exponential* type PDF
  5 $$f\left( {k;\eta ,\beta } \right) = C\eta^{1/\beta } {{\varGamma }}\left( {1 + \beta^{ - 1} } \right)^{ - 1} \exp \left\{ { - \eta k^{\beta } } \right\},\quad k > 0,$$
  (not to be confused with a Weibull PDF, see below), Iglesias and Pecharroman suggest deriving a formula for the *h*-index as the solution of the equation
  6 $$f\left( {x;\eta ,\beta } \right) = x.$$
  Interestingly, the solution may be derived in closed form (even if authors did not realize this) by means of the Lambert-*W* function. Unfortunately, this solution still depends on the value of an unknown free parameter, specifically $\beta$ [see their Eqs. (16) and (17)]. Hence, their formula could become a genuine estimator of the *h*-index—of the form $\hat{h} = \hat{h}\left( {C,T,T_{1} } \right)$, $r = 3$—only by constraining the unknown parameter $\beta$ to assume a fixed (but arbitrary) value $\beta_{0}$.

## A new formula for the *h*-index under the Weibull assumption

Let $N\left( y \right)$ be the empirical citation distribution function, i.e. the function giving the number of papers which have been cited $y$ times at most. Then, in particular, $n\left( y \right) = N\left( y \right) - N\left( {y - 1} \right)$, for $y = 1,2, \ldots$, $n\left( 0 \right) = N\left( 0 \right)$, is the number of papers that have been cited exactly *y* times. We assume that the citation rate of a paper is a random variable $X$ that is distributed as a two-parameter Weibull distribution, with CDF $F\left( {x;a,\beta } \right) = 1 - { \exp }\left\{ { - ax^{\beta } } \right\}$, $x > 0$, and 0 otherwise, where $a > 0$ and $\beta > 0$. The probability density function is then

7 $$f\left( {x;a,\beta } \right) = a\beta x^{\beta - 1} \exp \left\{ { - ax^{\beta } } \right\},$$

for $x > 0$, and 0 otherwise. The Weibull distribution is a rather flexible model: the PDF is reverse J-shaped for $\beta \le 1$ and bell-shaped otherwise.

Since our assumption involves a continuous distribution, a suitable discretization rule is needed. In particular, for every $y$, $y = 0,1,2, \ldots$, let $T\exp \left\{ { - ay^{\beta } } \right\}$ express the “expected” number of articles with at least $y$ citations. Hence, $\hat{n}\left( y \right) = T\int_{y}^{y + 1} {f\left( {x;a,\beta } \right){\text{d}}x = T \cdot \left( {F\left( {y + 1;a,\beta } \right) - F\left( {y;a,\beta } \right)} \right)}$ represents the expected number of articles with $y$ citations exactly, and $\hat{N}\left( y \right) = TF\left( {y + 1;a,\beta } \right)$ the expected number of papers which have been cited $y$ times at most. As a special case,

8 $$F\left( {1;a,\beta } \right) - F\left( {0;a,\beta } \right) = 1 - e^{ - a}$$

can be interpreted as a model for the so-called *uncitedness factor*, $\frac{{T - T_{1} }}{T} = \frac{n\left( 0 \right)}{T}$ (Hsu and Huang 2012; see also Egghe 2013; Burrell 2013). A Weibull model for the *h*-index is then yielded by the solution of the equation

9 $$T { \exp }\left\{ { - ax^{\beta } } \right\} = x,\quad x \in {\Re }\text{.}$$

Replacing $ax^{\beta }$ with $t$ in the equation, we have

10 $$te^{\beta t} = aT^{\beta } .$$

Thus, replacing $\beta t$ with $s$, we obtain the equivalent equation

11 $$se^{s} = a\beta T^{\beta } .$$

Hence, by definition of the above mentioned Lambert-*W* function, we find the solution $s = W\left( {a\beta T^{\beta } } \right)$ and, since $x = \left( {\frac{s}{a\beta }} \right)^{1/\beta }$, we finally arrive at the formula

12 $$x = \left( {\frac{{W\left( {a\beta T^{\beta } } \right)}}{a\beta }} \right)^{1/\beta } .$$

An empirical counterpart of the above theoretical model for the h index may now be obtained by substituting the parameters $a$ and $\beta$ with estimates, $a^{*}$ and $\beta^{*}$, based on suitable functions of the citation data only through the basic statistics $C,C_{1} ,T$ and $T_{1}$. This can be done firstly by using the uncitedness factor to derive the equation $1 - e^{ - a} = \frac{{T - T_{1} }}{T}$, that can be solved (under the assumption $0 < T_{1} < T$) for the variable $a$ as

13 $$a^{*} = \log \left( {\frac{T}{{T_{1} }}} \right),$$

as an estimate of parameter $a$, and secondly, by using the trimmed sample citation rate,

14 $$m^{*} = \frac{{C - C_{1} }}{T - 1} + 0.5,$$

as an estimate of the expectation of *X*, that is $E\left( X \right) = g\left( {a,\beta } \right) = a^{ - 1/\beta } {{\varGamma }}\left( {1 + \frac{1}{\beta }} \right) > 0$. Note that, by construction, our approximation slightly overestimates the true average number of citations, so that a correction for continuity by one-half is needed. We then find $\beta^{*}$ as the solution (method of moments) of the equation

15 $$m^{*} = g\left( {a^{*} ,\beta } \right),$$

that can be solved numerically. It should be noted that the existence and uniqueness of the solution of Eq. (15) are not always warranted a priori. Indeed, it can be proved that the necessary and sufficient condition for existence and uniqueness of the solution is $m^{*} > 1$ (see "Appendix"). We should then consider “out of range” the cases where $m^{*} \le 1$, and exclude them from the analysis.

With $a$ and $\beta$ replaced by $a^{*} = a^{*} \left( {T, T_{1} } \right)$ and $\beta^{*} = \beta^{*} \left( {C,C_{1} ,T} \right)$ in formula (12) one finally obtains ($r = 4$, ${\mathcal{S}} = \left\{ {C,C_{1} ,T,T_{1} } \right\}$)

16 $$\hat{h} = h_{WW} = \left( {\frac{{W\left( {a^{*} \beta^{*} T^{{\beta^{*} }} } \right)}}{{a^{*} \beta^{*} }}} \right)^{{1/\beta^{*} }} ,$$

where the suffix *WW* is motivated by the fact that the formula is based on a *Weibull* distribution and on the Lambert-*W* function.

## Analysis

### Two datasets

This section empirically investigates the effectiveness of formula $h_{WW}^{{}}$ as an estimate of the actual value of the *h*-index, $h$. We will compare estimates derived from $h_{WW}^{{}}$ with the real values of the *h*-index. In order to facilitate possible comparisons with other formulas (see below), we choose to use the same two datasets as in Bertoli-Barsotti and Lando (2017), where the authors present an empirical study based on citation data obtained from two different sets of journals belonging to two different scientific fields: (1) the *S&MM* list and (2) the *EE&F* list.

1. *S&MM list* The former dataset includes the 231 journals as selected from a former list of 568 journals identified as important (in the opinion of a group of experts) in the area “Statistics and Mathematical Methods” (S&MM). Overall, the S&MM dataset included 485,628 citations of 99,409 publications from these journals (for details see Bertoli-Barsotti and Lando 2017). For each journal, the actual value $h$ of the *h*-index was computed—on the basis of citations retrieved from the Scopus database in last week of December 2015—as the largest number of papers published in the journal between 2010 and 2014 and which obtained at least $h$ citations each, from the time of publication until December 2015. Thus, citation data referred to a 6-year citation window, 2010–2015, and a 5-year publication window, 2010–2014. The four basic statistics $C$, $C_{1}$, $T$ and $T_{1}$ were derived as well. The list of the 231 journals in the S&MM dataset is reported in Table 1.

**Table 1:** Basic statistics for the S&MM list of journals and the approximation of the Hirsch *h*-index calculated by means of the $h_{WW}$ formula (rounded values). The value $h_{WW}$ is not uniquely defined (N/D) for the first journal on the list (because of a too small average number of citations per paper). (Data retrieved in December 2015)

#	ISSN code	*C*	*C*_1_	*T*	*T*_1_	*h*	$\left\langle {h_{WW} } \right\rangle$
1	1405-7425	42	6	152	24	3	N/D
2	1012-9367	276	14	360	111	6	8
3	0017-095X	158	13	166	71	5	6
4	0315-3681	557	44	427	177	9	10
5	1081-1826	201	12	140	77	6	6
6	0957-3720	323	15	228	122	7	7
7	0002-9890	589	87	351	171	9	9
8	0361-0926	2033	28	1555	754	11	12
9	0117-1968	163	20	120	61	5	6
10	1210-0552	405	31	205	119	9	9
11	1056-2176	290	22	222	101	7	8
12	0165-4896	583	16	320	198	10	9
13	0315-5986	166	24	83	48	6	6
14	0736-2994	577	19	283	176	9	9
15	0399-0559	153	32	86	47	5	6
16	1303-5010	658	56	334	154	11	12
17	0927-7099	463	16	296	162	8	8
18	1351-1610	313	23	150	92	8	8
19	1292-8100	191	22	78	52	7	7
20	0361-0918	1036	45	635	369	9	10
21	0269-9648	263	16	172	84	7	8
22	1532-6349	308	15	141	93	7	8
23	0217-5959	522	33	261	155	9	9
24	1018-5895	424	25	189	115	9	9
25	0266-4763	2164	323	901	518	13	14
26	1471-678X	336	23	138	92	8	8
27	0304-4068	737	25	433	265	9	9
28	0020-7276	480	13	265	158	8	9
29	0023-5954	813	36	337	208	11	11
30	1220-1766	526	31	193	137	10	9
31	1226-3192	457	20	271	137	10	9
32	1618-2510	305	31	172	90	8	8
33	1083-589X	739	20	353	209	10	11
34	1048-5252	643	17	283	189	10	10
35	1004-3756	443	27	140	96	9	10
36	1009-6124	979	56	466	240	12	13
37	1120-9763	434	18	492	165	8	9
38	1369-1473	282	24	140	76	8	8
39	1230-1612	346	32	128	84	8	9
40	0026-1335	544	24	283	171	10	9
41	0218-348X	476	30	167	129	9	9
42	0167-7152	3169	40	1546	945	16	14
43	0032-4663	154	13	103	58	6	6
44	0282-423X	405	20	196	116	9	9
45	1748-670X	1933	36	822	543	14	13
46	0094-9655	1649	55	695	425	14	14
47	0039-0402	365	34	129	86	9	9
48	0894-9840	615	29	331	184	9	10
49	0398-7620	679	66	303	170	10	11
50	0219-0257	336	31	159	102	7	8
51	0319-5724	511	36	206	129	10	10
52	0020-3157	772	60	285	189	11	11
53	0898-2112	597	26	228	149	11	10
54	1524-1904	669	42	301	155	12	12
55	0963-5483	719	24	272	179	11	11
56	1547-5816	770	37	290	201	11	11
57	0001-8678	821	37	269	201	11	11
58	0021-9002	1168	35	477	321	13	12
59	0257-0130	719	18	260	179	11	11
60	1026-0226	2306	34	1036	610	15	15
61	0378-3758	3899	71	1334	907	18	18
62	0377-7332	1353	38	597	348	15	13
63	1560-3547	735	25	249	182	11	11
64	0893-4983	793	36	297	200	12	11
65	1387-5841	645	26	305	178	10	10
66	0167-6377	1702	33	582	399	14	14
67	1747-7778	837	294	135	93	10	12
68	1054-3406	1098	40	429	277	13	12
69	1619-4500	493	38	125	89	12	11
70	0143-9782	761	31	258	179	12	11
71	1432-2994	512	29	207	146	9	9
72	0219-4937	304	21	178	102	7	7
73	0033-5177	1734	42	878	522	14	13
74	1748-006X	779	31	238	184	11	11
75	1381-298X	364	23	113	82	9	9
76	0277-6693	825	61	217	160	14	12
77	1435-246X	735	43	263	175	11	11
78	1572-5286	587	25	158	114	12	12
79	1134-5764	458	59	246	128	8	9
80	0932-5026	829	26	396	210	11	12
81	0926-2601	769	78	286	196	10	10
82	0890-8575	333	47	119	74	8	9
83	0219-5259	803	32	254	179	12	12
84	0515-0361	447	37	150	89	11	10
85	0095-4616	626	46	192	135	11	11
86	0233-1934	1191	24	490	304	13	13
87	0167-5923	663	38	216	152	12	11
88	1469-7688	2100	77	653	404	17	18
89	1083-6489	1321	32	488	330	13	13
90	1392-5113	747	52	202	138	13	13
91	1863-8171	404	34	118	77	10	10
92	1380-7870	379	39	170	103	9	8
93	1862-4472	1866	32	652	438	15	15
94	0219-8762	905	65	300	185	15	13
95	0218-1274	5537	136	1370	1013	26	22
96	0747-4938	649	54	149	113	12	12
97	0020-7985	1280	28	417	268	16	15
98	0047-259X	3329	89	915	650	21	19
99	0303-6898	868	31	256	188	12	12
100	1471-082X	405	35	134	88	9	10
101	0924-6703	413	38	117	79	9	10
102	0346-1238	337	28	128	79	9	9
103	0748-8017	2076	31	534	380	19	18
104	1389-4420	793	124	184	124	15	13
105	0146-6216	737	30	215	155	12	12
106	0160-5682	3870	90	853	663	21	20
107	0960-0779	2712	118	570	443	20	19
108	0246-0203	1019	33	266	206	14	13
109	0306-7734	563	101	147	83	12	12
110	1350-7265	1499	40	375	294	15	15
111	0021-9320	910	22	274	207	12	12
112	0218-4885	1036	81	297	202	13	13
113	1945-497X	885	57	162	130	15	14
114	1352-8505	564	64	192	130	10	10
115	0003-1305	670	43	241	133	13	12
116	1076-2787	900	49	224	163	14	13
117	1862-5347	524	63	125	79	11	12
118	0022-4715	5302	91	1246	966	24	21
119	1133-0686	617	54	246	127	12	12
120	1539-1604	1075	183	286	194	13	13
121	1434-6028	7722	72	1849	1420	27	23
122	0304-4149	2652	44	791	577	15	16
123	0143-2087	1089	152	228	155	15	15
124	0323-3847	1221	129	327	230	15	14
125	0266-4666	1295	33	303	208	17	17
126	0925-5001	3452	61	849	611	22	20
127	1085-7117	682	49	183	129	13	12
128	0927-5398	1505	53	358	250	18	17
129	0899-8256	2942	76	696	512	20	19
130	0035-9254	1023	54	212	169	14	14
131	0893-9659	9519	95	1631	1295	35	30
132	0926-6003	2408	78	508	394	20	19
133	1368-4221	533	49	116	86	9	12
134	1386-1999	534	30	120	83	13	12
135	0254-5330	4505	190	1241	824	21	22
136	1180-4009	1611	52	325	236	18	18
137	0167-9473	7203	162	1541	1235	26	23
138	0013-1644	1350	78	262	214	16	16
139	1050-5164	2089	30	373	322	20	18
140	1544-6115	1073	56	260	199	15	14
141	1055-6788	1243	285	314	220	12	13
142	1076-9986	655	60	148	110	11	12
143	0025-5718	3127	60	595	488	22	20
144	0036-1410	3275	85	618	514	21	20
145	0740-817X	1881	44	382	302	18	18
146	0167-6687	2779	37	572	469	19	19
147	0364-765X	1237	61	227	180	17	16
148	1017-0405	2048	190	426	308	19	18
149	1369-183X	2904	90	469	398	24	21
150	1545-5963	3954	72	658	524	26	24
151	1064-1246	1887	40	813	504	16	14
152	0025-5564	2637	61	545	434	20	19
153	0036-1399	2359	63	466	390	19	18
154	0022-3239	4134	112	1005	685	24	23
155	0197-9183	1062	131	195	144	15	15
156	0949-2984	777	25	146	124	14	13
157	0178-8051	1744	47	408	313	17	16
158	1435-9871	1565	51	347	280	15	15
159	0091-1798	2227	56	408	353	20	17
160	0895-5646	742	43	123	103	13	14
161	0266-8920	1994	98	281	226	22	21
162	0363-0129	3796	112	661	534	25	23
163	0144-686X	1902	50	376	287	17	19
164	1061-8600	1661	73	290	237	18	18
165	1066-5277	3165	273	491	380	25	23
166	0020-7721	5586	180	1031	815	25	25
167	0303-8300	5093	124	1260	850	25	24
168	0006-341X	3854	75	717	565	24	23
169	0960-1627	854	36	189	149	14	13
170	0305-9049	886	56	209	157	12	13
171	0167-8655	12,864	1129	1417	1249	40	34
172	1932-8184	3207	74	648	414	24	25
173	1613-9372	832	36	171	134	13	14
174	1479-8409	461	46	115	74	11	11
175	1874-8961	1560	73	275	206	19	19
176	0960-3174	1891	109	408	284	19	19
177	1742-5468	3572	41	1564	950	19	16
178	0885-064X	1081	96	185	149	14	15
179	0007-1102	907	123	149	115	14	15
180	0171-6468	1499	82	215	165	17	19
181	1944-0391	484	28	201	81	11	12
182	1726-2135	1007	66	115	112	16	15
183	1544-8444	1703	56	242	210	17	19
184	0032-4728	558	34	101	87	11	12
185	0022-4065	752	34	113	88	14	15
186	0039-3665	913	176	158	119	13	14
187	0168-6577	536	53	93	80	12	12
188	0886-9383	2339	128	365	286	22	21
189	0018-9529	4175	94	469	387	29	29
190	1054-1500	5630	80	936	774	27	25
191	0304-4076	5332	165	723	609	30	27
192	0006-3444	2406	85	392	314	22	21
193	0964-1998	1287	50	234	177	17	17
194	1932-6157	2740	102	524	373	22	22
195	1468-1218	12,517	238	1271	1139	42	37
196	0025-5610	3997	194	567	442	27	27
197	1436-3240	3874	66	661	562	24	22
198	0167-6911	7259	351	731	617	37	35
199	0305-0548	13,373	156	1261	1135	45	40
200	0040-1706	1141	79	235	153	16	17
201	0165-0114	7962	108	1106	818	33	36
202	0883-7252	2055	108	286	234	22	21
203	0272-4332	6416	86	871	687	33	32
204	0277-6715	10,506	623	1780	1314	35	33
205	1568-4539	976	109	119	106	15	16
206	0022-2496	1417	82	199	160	19	19
207	0033-3123	1431	288	231	172	14	16
208	0951-8320	9529	95	926	850	37	34
209	0304-3800	13,918	412	1689	1511	36	33
210	1384-5810	2334	137	238	198	24	24
211	0169-7439	5880	187	726	645	30	27
212	1538-6341	1341	147	264	132	17	18
213	0030-364X	5098	120	554	487	30	29
214	0098-7921	1855	143	198	153	22	22
215	1465-4644	2347	142	304	253	23	22
216	0199-0039	1110	95	140	108	16	17
217	1052-6234	4321	765	414	345	25	28
218	0735-0015	1932	258	245	186	22	21
219	0167-9236	10,594	458	923	797	42	41
220	0162-1459	5231	156	663	519	31	31
221	0049-1241	803	148	115	99	14	13
222	0378-8733	2879	391	231	214	22	25
223	1470-160X	16,653	214	1636	1516	44	37
224	0070-3370	3714	74	420	376	26	26
225	0962-2802	1476	102	211	153	21	19
226	0090-5364	5835	315	486	433	31	34
227	0027-3171	1886	460	196	151	18	20
228	0883-4237	1909	375	237	151	21	21
229	1532-4435	14,005	966	1121	841	55	52
230	1369-7412	3186	475	169	149	23	29
231	1070-5511	1374	94	187	152	18	18
2. *EE&F list* The second dataset included the 100 journals (with a minimum number of 50 publications) top ranked according to the Scopus Impact per Publication (IPP; the IPP is defined as the ratio of citations in a year to papers published in the three previous years divided by the number of papers published in those same years) in 2014, within the Scopus subject area of “Economics, Econometrics and Finance” (EE&F). The citation data of all 100 journals in the EE&F list were retrieved during the last week of April 2016. The dataset obtained included 19,889 publications receiving a total of 74,096 citations. In this case, differently from the above dataset, in order to obtain citation and publication windows as similar as possible to those employed for the computation of the IPP 2014 by Scopus, the citations used were those received during 2014 of papers published within the previous 3 years 2011–2013 (for further details see Bertoli-Barsotti and Lando 2017). For each journal the actual value $h$ of the *h*-index was then computed as the largest number of papers published in the journal between 2011 and 2013 and which obtained at least $h$ citations each in the year 2014. The list of the journals in the EE&F dataset is reported in Table 2.

**Table 2:** Basic statistics for the EE&F list of journals and the approximation of the Hirsch *h*-index calculated by means of the $h_{WW}$ formula (rounded values) (Data retrieved in April 2016)

#	ISSN code	*C*	*C*_1_	*T*	*T*_1_	*h*	$\left\langle {h_{WW} } \right\rangle$
1	0022-0515	697	61	69	63	15	15
2	1531-4650	1161	58	127	117	18	18
3	1557-1211	1773	119	193	173	21	20
4	1540-6261	1529	54	190	178	17	18
5	0895-3309	995	44	133	111	15	17
6	1547-7185	1196	41	153	143	17	17
7	0092-0703	1015	111	140	128	15	15
8	0304-405X	2413	48	412	372	20	17
9	1468-0262	1014	35	187	171	14	13
10	1523-2409	434	26	81	71	10	11
11	1537-534X	483	56	92	79	10	11
12	1465-7368	1389	38	288	256	16	14
13	1540-6520	1062	52	175	147	15	16
14	1478-6990	795	38	155	140	13	12
15	1945-7790	516	22	113	103	10	10
16	0002-8282	3303	48	723	562	21	20
17	1945-7715	422	38	91	78	9	10
18	1741-6248	361	52	55	52	10	10
19	1469-5758	272	26	65	46	10	9
20	0165-4101	517	22	118	99	11	11
21	0925-5273	4678	92	1036	888	22	18
22	1542-4774	641	74	148	122	10	11
23	1537-5277	1086	24	234	213	12	12
24	0921-3449	1723	33	421	363	15	13
25	1467-937X	688	32	192	147	11	11
26	1945-774X	422	49	109	93	8	9
27	1873-6181	2683	26	667	565	16	15
28	1547-7193	948	56	213	188	13	12
29	1086-4415	324	36	57	49	10	10
30	1741-2900	234	34	54	42	8	8
31	1530-9142	1065	27	292	241	13	12
32	1530-9290	887	38	242	208	11	11
33	0001-4826	837	48	217	178	12	11
34	1090-9516	639	23	154	134	12	11
35	1547-7215	239	14	60	54	8	8
36	1941-1383	246	33	66	51	8	8
37	0921-8009	2620	34	675	567	17	15
38	0024-6301	248	33	58	44	9	8
39	1468-2710	586	36	142	122	10	10
40	1468-0297	760	29	210	179	10	10
41	1066-2243	355	27	85	73	9	9
42	1475-679X	398	21	111	86	10	10
43	0308-597X	1557	35	475	399	12	11
44	0022-1996	794	22	247	191	11	11
45	1096-0449	673	25	183	142	11	11
46	1573-6938	340	68	99	72	7	8
47	2041-417X	178	26	55	35	7	7
48	0306-9192	951	35	291	224	14	12
49	1537-2707	422	73	139	86	9	9
50	0013-0095	175	26	51	39	8	7
51	1052-150X	265	17	70	57	8	8
52	1533-4465	179	25	56	28	8	7
53	1526-548X	634	61	182	142	11	10
54	1873-5991	1725	22	540	426	13	13
55	1389-5753	231	17	64	56	8	7
56	1572-3089	268	24	86	71	7	7
57	1468-1218	2068	35	716	522	14	14
58	0304-3878	876	35	295	220	13	11
59	0047-2727	959	74	331	246	11	11
60	0969-5931	652	16	213	172	9	10
61	1532-8007	270	23	102	78	7	7
62	1075-4253	245	10	80	69	7	7
63	1386-4181	192	24	68	47	7	7
64	0265-1335	252	12	82	62	8	8
65	1537-5307	214	11	79	61	7	7
66	0301-4207	490	30	165	122	9	9
67	1096-1224	200	22	61	57	7	6
68	1467-6419	349	18	121	90	9	9
69	1932-443X	163	11	53	47	6	6
70	1756-6916	433	19	167	125	9	8
71	0304-3932	389	45	154	105	8	8
72	1572-3097	265	14	107	78	7	7
73	1464-5114	358	19	119	106	7	7
74	1911-3846	437	31	156	110	10	9
75	1096-0473	220	17	87	62	7	7
76	1095-9068	325	13	126	99	8	7
77	1389-9341	817	17	325	252	10	9
78	0217-4561	402	13	148	123	8	8
79	1548-8004	238	8	101	77	7	7
80	0304-4076	1037	28	404	305	12	10
81	0038-0121	218	38	74	49	7	7
82	0928-7655	340	38	133	93	8	8
83	1747-762X	205	38	91	60	6	6
84	1566-0141	273	16	110	87	7	7
85	1392-8619	368	45	117	79	9	9
86	1573-0913	719	18	261	198	11	10
87	1475-1461	244	26	83	64	8	7
88	1099-1255	372	15	163	113	8	8
89	0176-2680	416	18	179	135	7	8
90	1096-6099	242	25	113	78	6	7
91	1432-1122	175	8	89	64	5	6
92	0929-1199	553	28	244	172	8	9
93	1573-0697	2627	29	934	717	13	13
94	1467-0895	159	10	57	44	6	7
95	0378-4266	1993	36	893	621	13	12
96	1877-8585	167	15	64	50	6	6
97	1179-1896	272	9	127	88	6	7
98	0308-5147	231	14	88	60	8	8
99	1043-951X	449	19	194	145	8	8
100	0168-7034	176	13	74	41	8	7

### Estimation of the *h*-index with the formula $h_{WW}^{{}}$

Table 1 for the S&MM list and Table 2 for the EE&F list report, for each journal, identified by its ISSN code, the four basic statistics, $C$, $C_{1}$, $T$ and $T_{1}$, the *h*-index, $h$, as computed using the above procedure, and the value provided by the formula $h_{WW}^{{}}$ in its rounded-off version $\left\langle {h_{WW} } \right\rangle$, that is, in symbols,

17 $$\left\langle {h_{WW} } \right\rangle = \lfloor h_{WW} + 0.5\rfloor,$$

where $\left\lfloor { \cdot } \right\rfloor$ is the floor function (recall that the floor function of $x$ gives the greatest integer less than or equal to $x$). Note that, from an operational point of view, all estimating formulas (1) generate *real* numbers. However, for estimation purposes, these numbers should be rounded-off to the nearest integer, not only in order to produce numbers in the same range of values as the *h*-index but also to avoid “false precision”. (Hicks et al. 2015).

To give an example illustrating the calculation of this estimate, let us consider the case of the Journal of the American Statistical Association (ISSN 0162-1459, from the S&MM list). We have $C = 5231,C_{1} = 156,T = 663$ and $T_{1} = 519$. Hence

18 $$a^{*} = \log \left( {\frac{T}{{T_{1} }}} \right) = {\text{log }}\left( {663} \right) - {\text{log }}\left( {519} \right) = 0.2449$$

19 $$m^{*} = \frac{{C - C_{1} }}{T - 1} + 0.5 = \frac{5231 - 156}{663 - 1} + 0.5 = 8.166$$

Then, substituting $a^{*}$ and $m^{*}$ into the Eq. (15) we find

20 $$8.166 = \left( {0.2449} \right)^{ - 1/\beta } \varGamma \left( {1 + \frac{1}{\beta }} \right),$$

which yields the solution $\beta^{*} = 0.7365$. Thus, since

21 $$W\left( {0.2449 \cdot 0.7365 \cdot 663^{0.7365} } \right) = 2.26,$$

we finally conclude that

22 $$h_{WW} = \left( {\frac{{W\left( {a^{*} \beta^{*} T^{{\beta^{*} }} } \right)}}{{a^{*} \beta^{*} }}} \right)^{{1/\beta^{*} }} = \left( {\frac{2.26}{0.2449 \cdot 0.7365}} \right)^{1/0.7365} = 30.9,$$

so that the rounded-off version of $h_{WW}^{{}}$ in this case exactly coincides with the actual *h*-index, $h = 31.$

**Fig. 1:**
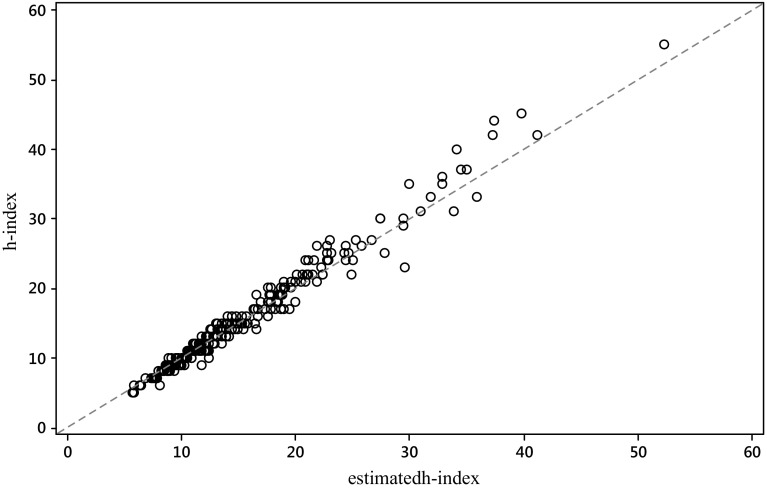
*Scatterplot* of the empirical value of the *h*-index *h* versus its predicted value by $h_{WW}^{{}}$, for the S&MM list of journals. The* dashed line* is identity, so ideally all the points should overlie this line

**Fig. 2:**
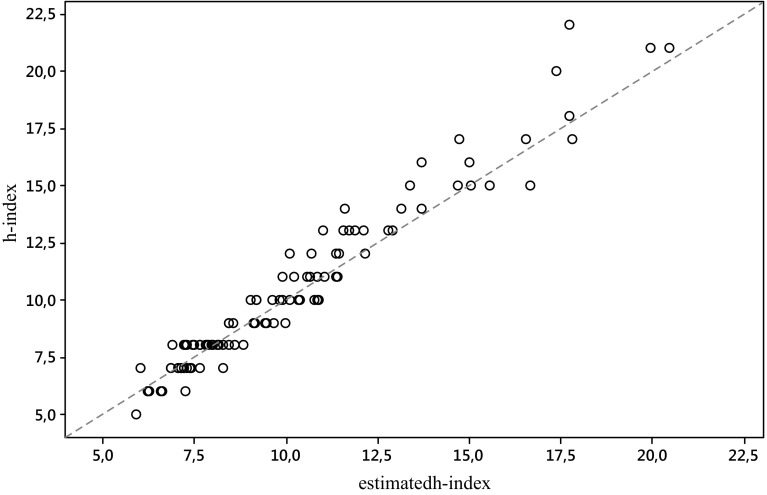
*Scatterplot* of the empirical value of the *h*-index *h* versus its predicted value by $h_{WW}^{{}}$, for the EE&F list of journals. The* dashed line* is identity, so ideally all the points should overlie this line

In Figs. 1 and 2 we plot for each journal, respectively for the S&MM list and the EE&F list, the empirical value of the *h*-index *h* versus its predicted value by $h_{WW}^{{}}$.

### A comparative analysis of the accuracy

To verify the accuracy of formula $h_{WW}^{{}}$, comparatively, we considered, among several possible ready-to-use formulas, the following ones among those defined above: $\tilde{h}_{W}^{\left( 1 \right)}$, $h_{SG} \left( {0.63} \right)$, $h_{SG} \left( {.75} \right)$, $h_{SG} \left( 1 \right)$, $h_{R}$, which have been viewed as important or promising alternatives to the $h_{WW}^{{}}$ formula—due to an empirically recognized high correlation with the *h*-index [see Bertoli-Barsotti and Lando (2017) for formula $\tilde{h}_{W}^{\left( 1 \right)}$, Glänzel (2006), Malesios (2015), Schreiber et al. (2012) and Schubert and Glänzel (2007) for formulas $h_{SG}$, and Redner (2010), for formula $h_{R}$]. To measure the magnitude of the observed accuracy, for each of the six estimation formulas respectively numbered as: (1) $h_{WW}^{{}}$, (2) $\tilde{h}_{W}^{\left( 1 \right)}$, (3) $h_{SG} \left( {0.63} \right)$, (4) $h_{SG} \left( {0.75} \right)$, (5) $h_{SG} \left( 1 \right)$, (6) $h_{R}$,

- we calculated the absolute relative error (ARE) of the estimator $\left\langle {\hat{h}_{j} \left( i \right)} \right\rangle$ of the actual *h*-index, $h_{j}$, for each journal $j$, $j = 1, \ldots ,J$,
  23 $${\text{ARE}}_{j} \left( i \right) = \frac{{\left| {\left\langle {\hat{h}_{j} \left( i \right)} \right\rangle - h_{j} } \right|}}{{h_{j} }},$$
  where $\left\langle {\hat{h}_{j} \left( i \right)} \right\rangle = \lfloor\hat{h}_{j} \left( i \right) + 0.5 \rfloor$ is the rounded-off version of formula $i$, $i = 1,2, \ldots ,6$, then,
- as a criterion with which to assess the overall quality of the formula, we computed the mean absolute relative error (MARE),
  24 $${\text{MARE}}\left( {\hat{h}\left( i \right)} \right) = \mathop \sum \limits_{j = 1}^{J} {\text{ARE}}_{j} \left( i \right)/J .$$

The results are summarized in Table 3.

**Table 3:** Relative accuracy, computed in terms of MARE, of different estimators of the *h*-index; *r* represents the number of basic metrics on which the estimation formula is based for each dataset, the smallest error is indicated by a boldface number

	$< h_{WW}^{{}} >$	$\left\langle {\tilde{h}_{W}^{\left( 1 \right)} } \right\rangle$	$\left\langle {h_{SG} \left( {0.63} \right)} \right\rangle$	$\left\langle {h_{SG} \left( {0.75} \right)} \right\rangle$	$\left\langle {h_{SG} (1)} \right\rangle$	$\left\langle {h_{R} } \right\rangle$
*r*	4	4	2	2	2	1
S&MM list (230 cases)	**0.060**	0.076	0.271	0.141	0.162	0.224
EE&F list (100 cases)	0.056	**0.050**	0.217	0.081	0.251	0.192

## Conclusion

This paper has addressed the need to gain better understanding of how simple citation metrics are related to the *h*-index, or rather, to a “good” proxy representation of the h index. This also responds to the more basic requirement of “building bridges” between different types of known and available measures of impact/impact indicators—under IIC.

Differently from other studies (that consider the problem of defining a “model” of the *h*-index), our concern has not been to estimate the parameters (sometimes even considered at the unit level, i.e. single journal, or single scientist; see e.g. Petersen et al. 2011) of a parametric model for the *h*-index under the assumption of knowing the entire citation pattern; rather, we addressed the quite different and more practical problem of finding a proxy representation of $h$ through a universal formula that only depends on few summary statistics of the data. The formula $h_{WW}^{{}}$ is “universal” in the sense that it gives a proxy representation of *h* that holds for any given journal and any dataset.

The issue of determining an indicator under IIC is closely related to the search for a solution of the problem of recovering and comparing impact indicators from different databases. As a simple but significant example of this issue, we may cite the specific problem of determining/estimating the IF for journals using the Google Scholar-based *h*-index as a predictor (Bertocchi et al. 2015).

As confirmed in our case study analysis, the *h*-index can be viewed as an almost-exact function of $C,C_{1} ,T$ and $T_{1}$, through $h_{WW}^{{}}$, i.e. that the basic statistics $C,C_{1} ,T$ and $T_{1}$ provide salient information for the evaluation of the *h*-index with high precision. In practice, while computation of the *h*-index *h* requires knowledge of the entire citation profile (or at least large part of it, e.g. the so-called *h*-core), formula $h_{WW}^{{}}$ requires knowledge of only a few elementary summary statistics, but reproduces the actual value of *h* quite well. In truth, in our computations we found that the estimates yielded by $h_{WW}^{{}}$ were slightly biased downwards for quite high values of the *h*-index but, as can be seen from Table 3, overall the formula $h_{WW}^{{}}$ yields very accurate approximations to the empirical value of the *h*-index, with values of the MARE ranging around 5–6%, not too dissimilar from those obtained by formula $\tilde{h}_{W}^{\left( 1 \right)}$ (Bertoli-Barsotti and Lando 2017). Both formulas $\tilde{h}_{W}^{\left( 1 \right)}$ and $h_{WW}^{{}}$ exhibit comparable levels of accuracy (the advantages of the formula $\tilde{h}_{W}^{\left( 1 \right)}$, as compared to formula $h_{WW}^{{}}$, may be that: (i) it yields an *explicit* expression of the basic indicators $C,C_{1} ,T$ and $T_{1}$, while the latter not, and (ii) it is based on a simpler probabilistic model). Even though the Pearson correlation, $\rho$, is *not* an adequate measure of the accuracy of the estimation and should not be used to compare the effectiveness of the different estimators considered (and this is the reason why this concept has been banished from this study), for the sake of completeness we point out that: (1) for the S&MM dataset (230 journals), we found $\rho \left( {h,h_{WW}^{{}} } \right) = 0.99$, $\rho \left( {h,\tilde{h}_{W}^{\left( 1 \right)} } \right) = 0.98$, $\rho \left( {h,h_{SG} } \right) = 0.98$ and $\rho \left( {h,h_{R} } \right) = 0.96$; (2) for the EE&F dataset we found $\rho \left( {h,h_{WW}^{{}} } \right) = 0.97$, $\rho \left( {h,\tilde{h}_{W}^{\left( 1 \right)} } \right) = 0.98$, $\rho \left( {h,h_{SG} } \right) = 0.97$ and $\rho \left( {h,h_{R} } \right) = 0.90$. Ultimately, despite the differences between the datasets considered—in terms of scientific areas, time windows for publication and citation, types of “citable” documents considered, mean level of the basic indicators $C,C_{1} ,T$ and $T_{1}$ (with values of respectively 2111, 95, 432 and 312 for the S&MM dataset and 741, 33, 199 and 159 for the EE&F dataset)—we may conclude that, on the whole, $h_{WW}^{{}}$ provides fairly accurate approximations to the real value of the *h*-index, at least for not too large values of *T* (e.g. $T < 2000$), *m* (e.g. $m < 20$) and *h* (e.g. *h* < 40), such as those considered in this study.

## Appendix

### Conditions for existence and uniqueness of a solution of Eq. (15)

For every fixed $a = a^{*} > 0$, $g\left( {a^{*} ,\beta } \right) \to + \infty$ as $\beta \to 0$ and $g\left( {a^{*} ,\beta } \right) \to 1$ as $\beta \to + \infty$. Moreover, since

25 $$\frac{\partial }{\partial \beta }g\left( {a^{*} ,\beta } \right) = \frac{{g\left( {a^{*} , \beta } \right)}}{{\beta^{2} }}\left( {\log a^{*} - \psi \left( {1 + \frac{1}{\beta }} \right)} \right),$$

where $\psi$ is the digamma function, i.e. the function defined by $\psi \left( z \right) = \frac{\text{d}}{{{\text{d}}z}}\log \varGamma \left( z \right) = \varGamma '\left( z \right)/\varGamma \left( z \right)$ (see Johnson et al. 2005, pp. 8–9), we find that the inequality

26 $$\frac{\partial }{\partial \beta }g\left( {a^{*} ,\beta } \right) < 0$$

holds if and only if it holds

27 $$\log a^{*} - \psi \left( {1 + \frac{1}{\beta }} \right) < 0.$$

Now, the function $\psi \left( {1 + \frac{1}{\beta }} \right)$ is (convex and) strictly decreasing from $+ \infty$ at 0 to

28 $$\mathop {\text{lim}}\limits_{\beta \to \infty } \psi \left( {1 + \frac{1}{\beta }} \right) = \psi \left( 1 \right) = \varGamma^{\prime}\left( 1 \right) = - \gamma ,$$

where $\gamma$ is the Euler–Mascheroni constant ($\gamma = - {{\varGamma^{\prime}}}\left( 1 \right) \cong 0.5772$), at $+ \infty$.

Hence $\psi \left( {1 + \frac{1}{\beta }} \right) > - \gamma > \log a^{*}$ for every $\beta > 0$ if and only if $0 < a^{*} \le \exp \left( { - \gamma } \right) \cong 0.561$.

Thus the following two cases are possible.

- If $0 < a^{*} \le \exp \left( { - \gamma } \right)$, the inequality (26) holds. In this case the function $g\left( {a^{*} ,\beta } \right)$ is strictly decreasing from $+ \infty$ at 0 to 1 at $+ \infty$, with a limit approached from above. We conclude that, in this case, Eq. (15) has a unique solution if and only if $m^{*} > 1$; otherwise, if $m^{*} \le 1$, Eq. (15) has no solution.
- On the other hand, if $a^{*} > \exp \left( { - \gamma } \right)$, the derivative function $\frac{\partial }{\partial \beta }g\left( {a^{*} ,\beta } \right)$ changes its sign from negative to positive at $\beta = \beta_{0}$, for some $\beta_{0} > 0$; hence $g\left( {a^{*} ,\beta } \right)$ is strictly decreasing for every $0 < \beta < \beta_{0}$, and strictly increasing for every $\beta > \beta_{0}$, and the point $\beta_{0}$ is a global minimum for $g\left( {a^{*} ,\beta } \right)$. Moreover since, as seen before, $\mathop {\lim }\limits_{\beta \to \infty } g\left( {a^{*} ,\beta } \right) = 1$, then $0 < g\left( {a^{*} ,\beta_{0} } \right) < 1$, and the limit at infinity is approached from below. We conclude that, in this case too, Eq. (15) has a unique solution if and only if $m^{*} > 1$; conversely, if $m^{*} \le 1$ Eq. (15) may have two solutions, or no solution at all.

In both cases (a) and (b), Eq. (15) has one and only one solution if and only if $m^{*} > 1$.
